# The role of incline, speed and work rate on the choice of technique in classical roller skiing

**DOI:** 10.1371/journal.pone.0236102

**Published:** 2020-07-15

**Authors:** Johannes Løkkeborg, Gertjan Ettema

**Affiliations:** Centre for Elite Sports Research, Department of Neuromedicine and Movement Science, Faculty of Medicine and Health Sciences, Norwegian University of Science and Technology, Trondheim, Norway; Universita degli Studi di Verona, ITALY

## Abstract

Cross-country skiers use different sub-techniques like ‘gears’ (diagonals stride, double poling with kick, and double poling) depending on terrain (incline) and demand (speed and external work rate). Previous studies have identified the major effect of speed and incline, but not any potential interaction between these parameters: the incline-speed combination determines the work rate, which in itself may be a controlling factor. The aim of this study was to investigate the role of these task conditions (external work rate, speed, incline) and their interactions on the choice of sub-technique in classical roller skiing. Twelve male and nine female cross-country skiers executed three subsets of protocols in which two of three condition parameters were altered every 15 seconds while roller skiing on a treadmill. This design created a quasi-random set of combinations of speed, incline and work rate that were analysed on sub-technique choice. A repeated measures model with sex as between subject factor was conducted for each subset of protocols. The incline appeared to be the factor affecting sub-technique choice most, but not exclusively; at constant incline, athletes applied different sub-techniques, depending mostly on speed rather than work rate. Most athletes did not use one particular sub-technique for a given speed-incline-work rate combination, but it depended on the protocol and direction of condition change in the constant speed protocol (hysteresis). Only minor differences between men and women existed regarding the impact of condition factor on sub-technique choice. The findings disagree with the notion of a simple mechanism that explains the choice of sub-technique, but rather opt for a complex structure that entangles various mechanisms which play a role in the choice of sub-technique under moderate effort conditions.

## Introduction

Competitive cross-country skiing is performed in either freestyle (skating) or classical style using different clearly identifiable sub-techniques to adapt to varying terrain and conditions. Utilization of these sub-techniques has previously been described as a ‘gear’ system, where the athletes choose sub-technique based on factors such as terrain and speed [1]. The classical style primarily consists of three different sub-techniques, double poling (DP), double poling with kick (DK) and diagonal stride (DIA). In DP, which is the highest gear applied at high speeds on flat terrain, only the poles are used directly for propulsion, in a synchronized manner. Although all propulsion is provided through the poles, the legs contribute considerably to this indirectly [2–4]. In contrast to DP, DIA is employed in steeper terrain at lower speed and in which a diagonal coordinated pattern is applied as in walking and running. In DIA, propulsion is provided through both skis and poles with approximately 22% and 42% of the cycle time being used through ski and pole, respectively. Nevertheless, most propulsive power is provided via the skis [5, 6]. In DK, the arms are used in a similar way as in DP, but the sub-technique also includes an alternating leg kick. The leg kick provides propulsion via one ski in-between each poling phase and results in longer cycle times and a higher duty factor (i.e., longer relative propulsion phase) compared to DP [1, 7]. DK is mostly used on slightly uphill terrain, in-between the DP and DIA sub-techniques. Changes between these sub-techniques occurs often during races. For example, it was found that the national level athletes on average changed between the different sub-techniques almost 300 times during a 10 km classical race [8].

As mentioned, it is well known under which conditions these different sub-techniques in general are used. However, there is no consensus regarding which factor triggers sub-technique changes, and to what extent task conditions (i.e., incline, speed and work rate) determine the choice of sub-technique. At constant speed, consistent changes from DP to DK and further from DK to DIA with increasing incline are found [9, 10]. Furthermore, a larger part of the athletes prefers DP at higher speed on a constant incline [10]. Considering that, in these studies, the external work rate also changed with change of incline or speed, one cannot separate the effect of speed or incline from that of work rate. This issue was circumvented by conducting protocols where both incline and speed changed simultaneously, but such that external work rate was constant [11]; the incline, not speed, at which athletes changed sub-technique was almost identical at the different work rates this protocol was executed at. This indicated that incline rather than speed is the main factor determining choice of sub-technique. Yet, since external work rate was kept constant during the exercises, the effect of work rate on choice of sub-technique is still unknown.

Several mechanisms that explain the choice of sub-technique have been proposed and include factors such as efficiency, instability, force limitation, power fluctuations, and subjective feelings of comfort [9–13]. However, it is uncertain which of these mechanisms explains the choice of sub-technique. Of course, it is possible that several mechanisms play a role in this choice. Recently, it was found that exhaustive exercise had no impact on choice of sub-technique, and it is thus unlikely that physiological factors play a significant role [14]. There may exist a force limit for the poles that an athlete would not wish to exceed [10]. With increasing incline, sub-technique changes would occur in advantage of a sub-technique where less force is provided via the poles, i.e. DK and DIA [15]. In addition, during DP at steep incline, lower body contribution might be limited due to lesser movement perpendicular to the ground surface and thus reducing the use of the body’s potential energy fluctuations [11, 13]. This would in turn place even higher emphasis on the upper body for propulsion at steep incline [13]. In addition, leg thrust time is reduced depending on speed when using DIA [10]. A minimum feasible thrust time of 0.1 was proposed and that thrust time below ~0.22 s would trigger a sub-technique change allowing for longer thrust times at that same speed. This notion was supported by findings in Dahl et al. [13], but not confirmed in Ettema et al. [11] who found athletes change between sub-techniques at different speeds, depending on work rate and incline.

General measures of upper- and lower-body power have previously been shown to be related to DP and DIA performance [15]. Thus, upper- and lower-body power and strength may of course influence individual sub-technique preferences. In that case, this can also help explain the relatively large individual differences in sub-technique preferences found in previous studies [9–11].

Since none of the studies on choice of skiing sub-technique has unequivocally revealed what task factor (incline, speed, work rate) determines this choice, we aimed to further investigate this issue by extending the protocol employed in one of our previous studies [11]. By performing and comparing protocols in which either external work rate [11], incline or speed [10] is kept constant and the other two are changed accordingly, this allowed us to investigate, in more detail than the previous studies, to what extent these factors influence the choice of sub-technique in classical cross country skiing. Additionally, we investigated if individual differences in choice of sub-technique (i.e., preference) can be explained by differences in upper- and lower body power.

Based on previous findings in Ettema et al. [11] we hypothesized that, under moderate effort conditions, incline is the primary determining factor for the choice of sub-technique and that athletes with higher upper-/lower-body power capacity would show increased use of DP, due to the higher reliance on the upper-body for propulsion in DP compared to DIA [15].

## Methods

### Participants

12 male (179.6 ± 5.9 cm, 72.4 ± 4.7 kg, 21.2 ± 4.0 yrs) and 9 female (171.6 ± 4.3 cm, 65.0 ± 6.6 kg, 20.4 ± 2.1 yrs) cross-country skiers volunteered to participate in the study. The participants were junior and senior competitive skiers and were familiar to roller-skiing on a treadmill. Written informed consent was obtained from all participants, and they were informed that they could withdraw from the study at any point. The procedures were verbally explained to each athlete before testing. This study is outside the mandate of Regional Committees for Medical and Health Research Ethics (Norway), i.e., medical and health research, and therefore registered, assessed and approved by Norwegian Social Science Data Services (NSD). The study was conducted in accordance with the Declaration of Helsinki.

### General experimental design

Testing was done over two days. On the first day, the participants performed two power tests: a bench pull (for upper body, UB) and a leg press (for lower body, LB). On the second day, nine different protocols with classical roller-skis on a treadmill were completed. Treadmill roller-skiing was chosen in order to minimize environmental influences and thus achieve standardized conditions. All experiments were performed at the core facility NeXt Move, Norwegian University of Science and Technology (NTNU).

### Power testing (bench pull and leg press)

As a warm-up before the power tests all participants performed ten minutes of running at low intensity. The participants were placed in standard position (see below) and instructed to pull (bench pull) or push (leg press) as forcefully as possible during each test. All participants performed the leg press exercise before the bench pull with ample rest between each test. A constant order was preferred because any systematic bias that may have been introduced this way would be nullified in statistical testing.

The leg press [16, 17] was performed in a Keiser leg press (Air300 Leg Press, Keiser Corporation, California, USA) and a standardized ten-repetition test was applied. Based on the participants estimated 1RM, the apparatus estimated ten loads with increasing resistance. On the first load the participants performed two attempts to get familiar with the apparatus before the ten-repetition test started. On the forthcoming loads, only one attempt was performed. The rest time between attempts was also set by the apparatus and increased as the load increased. The test was performed until the participant was not able to complete one repetition. Thus, if the estimated 1RM was too high, the test ended with fewer than ten attempts, and conversely continued if attempt number ten was successfully performed. The seat was adjusted such to obtain a 90° knee angle at the starting position and the press action ended with extended knees in ≈180° angle. Maximum lower-body power was identified as the single highest power achieved amongst all the lifted loads.

The bench pull [18, 19] was performed lying on a bench in prone position, with an Olympic weight lifting bar of 20 kg (Leoko, Tampere, Finland). Power testing was performed with at least 3 sets of 3 repetitions with increasing weight to obtain a valid estimate of the participants’ force-velocity relationship and maximal power. If a reduction in power was not evident on the third set, higher loads were lifted. The test started with a 20 kg load (lifting bar only), and the weights were increased in intervals of 5 kg for women and 7.5 kg for men. On the first load, the participants performed two sets in order to get familiar with the test. The participants could rest for 2–3 minutes between each set. The starting position was with fully extended elbows. The bar was held with a prone grip at shoulder width between hands. The pull ended when the bar touched the bench with flexed elbows. If the bar did not touch the bench or the elbows were not properly extended before the lift, the attempt was not approved. A linear encoder (MuscleLab, Ergotest Innovation A.S., Porsgrunn, Norway) was attached to the bar, and the included software calculated power according to:

P=m*(−g+a)*v

where *m* is the mass of the lifted weight, *g* is the gravitational acceleration (-9.81), *v* the velocity, and *a* the acceleration of the bar. Maximal power was estimated by the software as the highest averaged power produced during the upward phase of a repetition.

### Roller-ski testing

The classical roller-skiing was performed on a 5x3 meter motorized treadmill (Forcelink Technology, Zwolle, The Netherlands) using a safety harness. All participants used the same pair of IDT roller-skis (IDT sports, Lena, Norway) equipped with wheels of rolling resistance category 2. The athletes could use their own pair of poles with self-preferred length. Before performing the nine different protocols, all participants performed a ten-minute warm up on the treadmill, with low to moderate intensity at varying speed and incline to ensure that all sub-techniques were used. The participants were instructed that they could use the sub-technique that they felt was most natural and were free to change sub-technique at any time during the protocols. They were also aware of the length of the protocols and that speed and/or incline would change during the tests.

**Fig 1:**
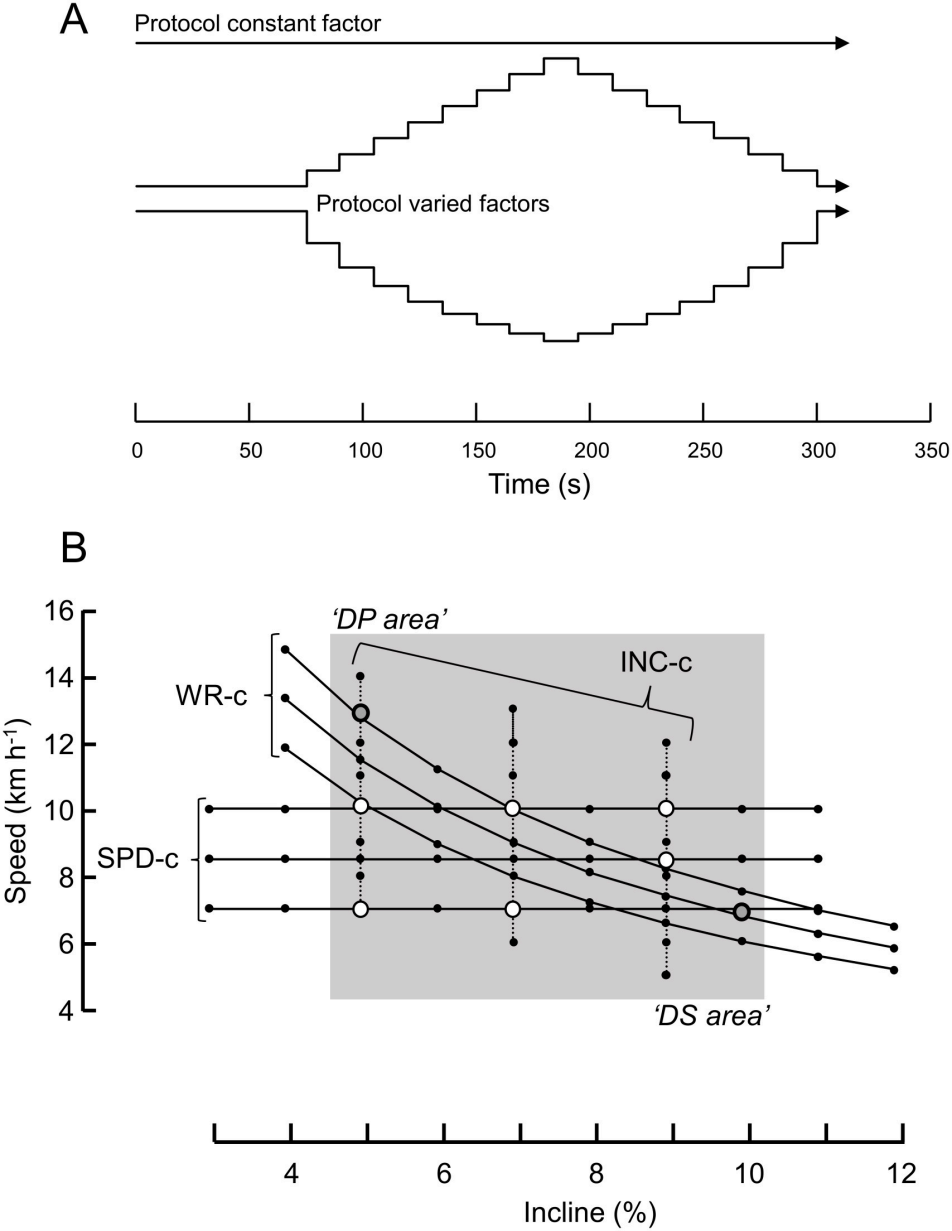
Description of the main protocol. A: The general protocol design depicting the progression of the three condition variables (incline, speed, and work rate) of which one is kept constant. B: The combination of incline and speed for the three subsets and levels. Black markers indicate the condition combinations applied in the protocols. Cross-points that were compared to identify any history effect (through what combination of conditions, i.e., protocol, the condition of interest was attained) are indicated by larger white markers. Two cross-points at the outer range of incline-speed combinations (at which one sub-technique was expected; ‘DP-area’ (double poling) and ‘DS area’ (diagonal stride)) are marked grey. WR-c, SPD-c, and INC-c are constant work rate, constant speed and constant incline protocol, respectively.

The different combinations of speed and incline during the protocols were chosen based on previous studies [9–11]. They were also designed with an intensity that was expected to be sub-maximal in order to avoid fatigue during the testing [11], i.e., the highest power for a 15 second period not exceeding 300 Watts, the average per exercise not higher than 200 Watts. Except for duration, the main set-up was very similar as in our previous study [11] and is depicted in Fig 1A. All protocols consisted of an ‘initial order’ and a ‘reversed order’ to investigate the effect of direction of change (hysteresis). After a 60 s initial condition period during which the athlete could adjust to the settings, a period followed during which two of three factors were changed every 15 seconds. The combination of nine protocols allowed us to not only investigate if any condition solely determines choice of sub-technique, but also if the history of how one attains that condition affects the sub-technique choice. All combinations of speed, incline and external work rate are shown in Fig 1B: the nine protocols consisted of three subsets in which one of three condition parameters (incline, speed, work rate) was held constant, while the remaining two were changed accordingly. In each subset, the test was repeated at three different levels of the constant parameter. Thus, three tests were performed at three constant external work rates with varying speed and incline (subset WR-c), three tests at three constant inclines and varying speed and power (subset INC-c) and three tests at three constant speeds and varying incline and power (subset SPD-c). The protocols were performed with identical speed and incline for all participants. Thus, the relative work rate (Watt kg^-1^) was close to identical between participants. The protocols were quasi-randomized in order and grouped in three bouts, each consisting of one protocol from each subset. In addition, the first bout started with a protocol with the lowest work rate from one of the subsets. This was done to ensure that the participant was properly warmed-up and to minimise a possible influence of protocol order. Of all possible order combinations, 7 3x3 order-matrices were constructed and each assigned to three participants by blindfolded drawing. Between each bout, the participants could rest for five minutes.

Work rates in subset WR-c were 2, 2.25, and 2.5 Watt kg^-1^ body mass. Total external work rate (*P*_*t*_) and required speed (*v*) to obtain a particular work rate for a given incline (α) was estimated as previously [11] the sum of power against gravity (*P*_*g*_) and power against rolling friction (*P*_*f*_)

$$P_{t}=mg\;v\;\left(sin\alpha +\mu \;cos\alpha \right)\;↔\;v=P_{t}\;{\left(mg\;\left(sin\alpha +\mu \;cos\alpha \right)\right)}^{-1}$$

Where *m* is the system mass (i.e., participant’s body mass plus equipment), *g* is the gravitational acceleration, *θ* is the angle between the horizontal plane and the incline of the treadmill, *μ* is the rolling friction coefficient, and *v* is the velocity of the treadmill in ms^-1^. The rolling friction coefficient (*μ*≈0.18) was determined by a towing test [20] after warming up to avoid changes by temperature during the test [21]. It must be noted that the external work rate does not reflect the total muscular work rate because of internal losses that may differ between techniques and conditions [22]. Also, the total physiological load (metabolic rate), which depends on (variable) gross efficiency is not reflected. Identification of sub-techniques was done according to Ettema et al. [11] based on quantifying the continuous phase between movement of the skis. For this purpose, a reflective marker was placed on the side of each ski, just in front of the back wheel. Motion capturing was performed at 100 Hz using six cameras (Oqus 3D motion capture system, Qualisys AB, Gothenburg, Sweden). The continuous phase recordings were synchronized with the treadmill protocol using two reflective markers attached on the treadmill base at 1 m inter-distance; these markers allowed quantification of the treadmill’s incline at all time in the same recording. In the INC-c protocol, the belt speed (calculated from the pole speed in the periods of belt contact, reflective markers attached near the bottom of the poles) was used for this purpose.

### Statistical analysis

In situations where two different sub-techniques were used for a given constant condition (15 seconds), the sub-technique used for the largest part was chosen for later analysis. A repeated measures model (SPSS 25; IBM Corp., Armonk, NY, USA) with sex as between subject factor was conducted. Each protocol subset (with three levels as the within-subject factor) was treated separately in the analysis. The outcome from the ANOVA was the minimum and maximum incline, speed, and work rate (depending on subset) at which the DK sub-technique was used. This result was used as identifier for DS-DK and DK-DP shifts. Data were checked for normality using a Shapiro-Wilk test. If assumption of sphericity was not met, Greenhouse-Geisser correction (when epsilon <0,75) or Huynh-Feldt correction (when epsilon >0,75) were used for significance assessment. Absence of a statistical effect of the constant parameter in each subset on any of the two varying parameters would indicate a role of that varying parameter in the choice of sub-technique. For example, if in the WR-c subset, athletes would change sub-technique at the same speed (i.e., speed is the controlling factor), but different incline for each of three work rates (work rate being the constant parameter), this would show as a non-significant effect of work rate on speed-of-shift and a significant effect on incline-of-shift. Small differences in speed between protocols at WR-c (speed was based on a given preset incline) occurred, giving artificial disfavor for not finding ‘no significance for speed-of-shift’ [11]. Therefore, rounded speed values were used in the statistical analysis, but exact values are presented in the results (see Ettema et al. [11] for details).

The three protocols had various cross-points, i.e., speed-incline combinations that were (almost) identical for two or three protocol types. To investigate if previous activity played a role in sub-technique choice, the subset outcomes were compared at eight of these cross-points (Fig 1B). Binomial statistics were used to estimate the confidence interval (C.I.) for proportion of athletes who used the same sub-technique at a given speed-incline combination irrespective of the preceding condition (history of protocol) and those who chose different sub-techniques.

Hysteresis effects, i.e. any difference of sub-technique choice depending on the direction of protocol, maximal incline of DP and DK on the initial (increasing incline) and reversed order (decreasing incline) were compared using a paired *t*-test. Only the protocols with changing incline were chosen because of the expected primary influence of incline on sub-technique choice. An individual mean of the three protocol levels (for both WR-c and SPD-c) were used in favour of a complete mixed model analysis due to a lacking number of individuals employing all sub-techniques in all protocols.

A Pearson´s correlation analysis was used to investigate the relationship between DP and DS use and upper and lower body power. Men and women were compared using an unpaired two-sample *t*-test.

Statistical significance was determined based on a significance level of p<0.05.

## Results

### Influence of speed, incline, and work rate

For all protocols, a very clear pattern of sub-technique choice during the protocol evolvement was seen in almost all cases, from DP to DK to DS, or vice versa, in which athletes showed distinctive shifts between sub-techniques. Thus, the approach described in the statistics section, i.e., using periods in which DK was used to identify sub-technique shifts, could be applied.

**Fig 2:**
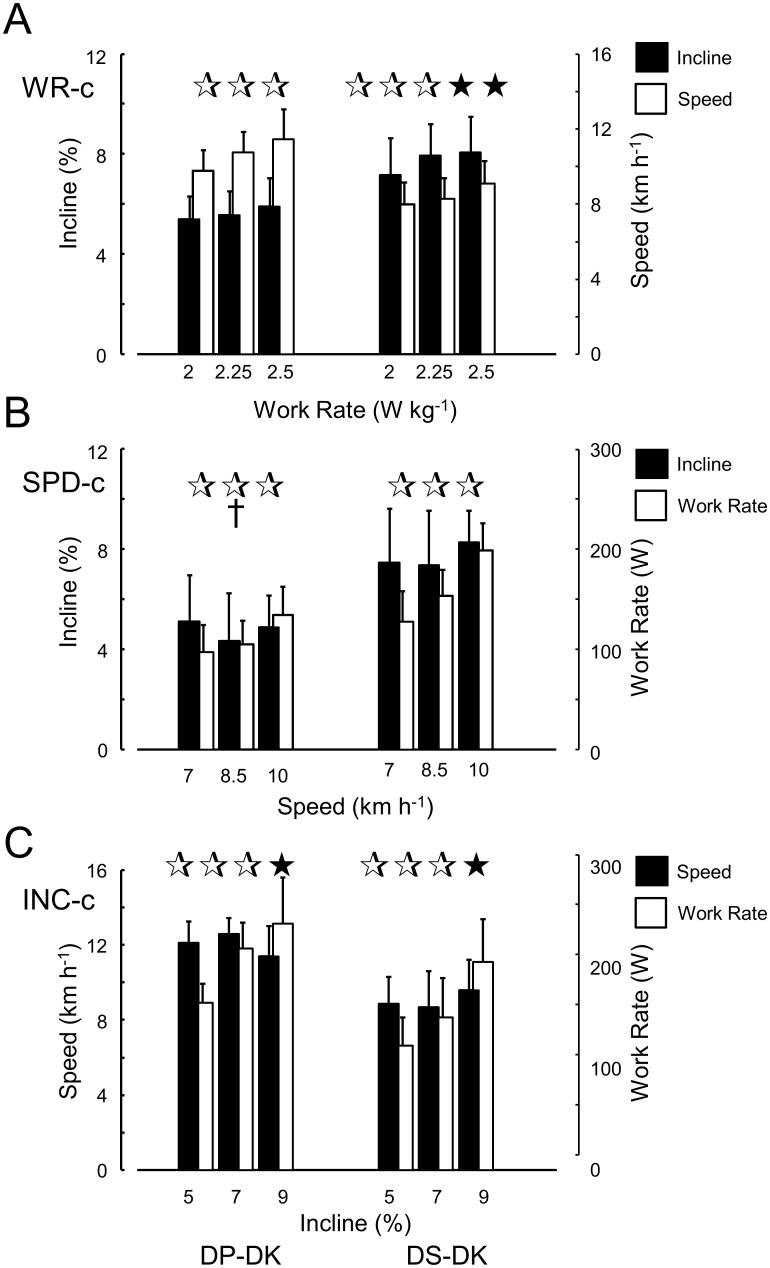
Incline, speed and work rate at sub-technique changes between double poling and double poling with kick (DP-DK) and between diagonal stride and double poling with kick (DS-DK). Separate findings are shown for each of three protocol subsets (Fig 2A-2C). Asterisks indicate significance (three: p<0.001; two p<0.01; one: p<0.05). The colour code (black-white) of the asterisk indicates the variable of interest, † indicates sex interaction.

In WR-c, the speed-of-shift for both sub-technique shifts was affected by work rate (p<0.001), the incline-of-shift only for DS-DK (p = 0.001). In SPD-c, the work rate-of-shift was affected by speed for both cases (p<0.001), while the incline-of-shift was not. Note that these findings indicate that speed is not a sole control variable, and neither is incline for the DS-DK shift in WR-c. Incline likely is a control variable in the other conditions. Significant sex effects were identified in SPD-c (DP-DK) (p = 0.01). Post analysis revealed that women behaved differently from the general outcome regarding the incline of DP-DK shift which was significantly affected by speed (p = 0.022). The statistical outcome for men was the same as for both sexes combined. In INC-c, the work rate of sub-technique shifts depended on incline (p<0.001), while the speed-of-shift did not (p = 0.058 DP-DK; p = 0.159 DS-DK), indicating that work rate is not a control variable, and speed likely is (Fig 2).

**Fig 3:**
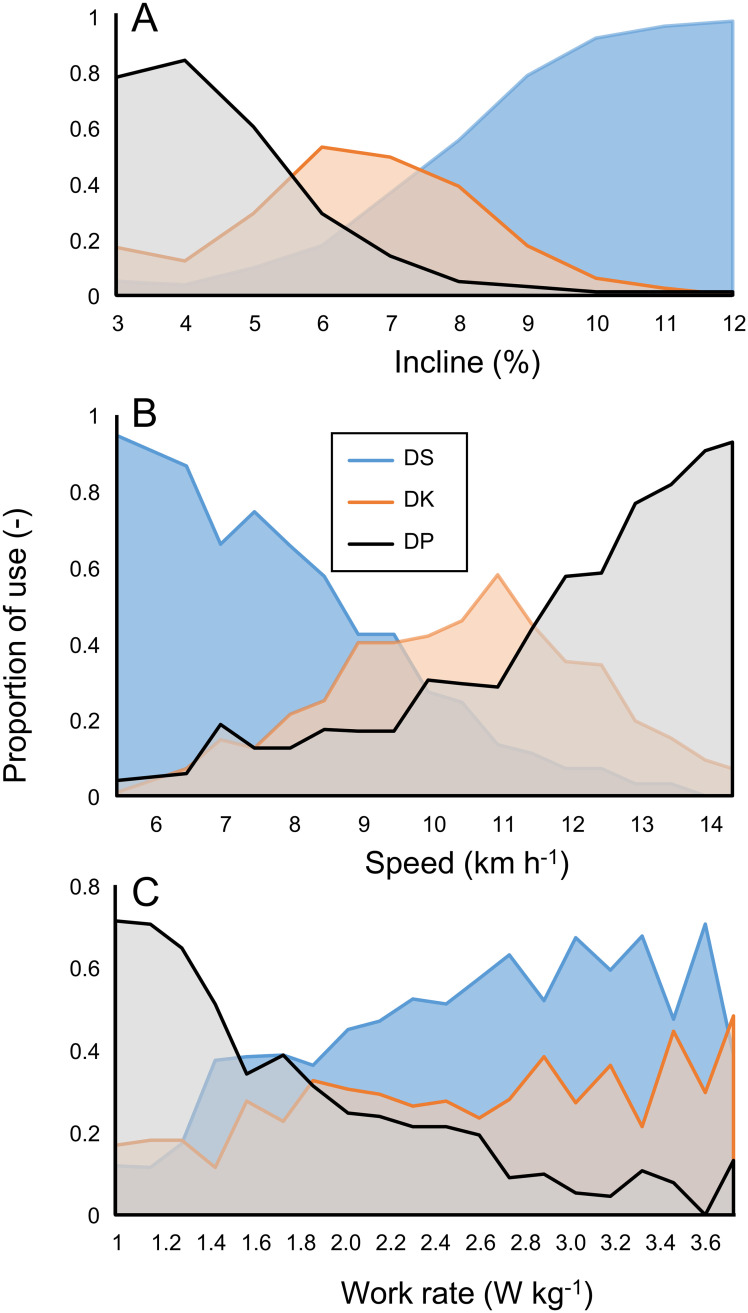
Proportional use of sub-techniques. Mean proportional use per individual are shown as function of incline (A), speed (B) and work rate (C). Data are based on all protocols. DS: diagonal stride, DK: double poling with kick, DP: double poling.

At intermediate inclines and speeds, many athletes tended to use all three sub-techniques depending on the protocol, i.e., combination of speed, incline, and/or work rate (Fig 3). At the boundary values of incline and speed, the choice of sub-technique was almost uniform. This hardly applied for work rate (Fig 3 bottom diagram). Two extreme cross-points (Fig 1B) where either DP or DS was anticipated, showed indeed that the vast majority (18–19) of athletes used the expected sub-technique in all protocols. Two athletes used the same sub-technique, independent of protocol, at all the cross-points, i.e., (close to) identical speed-incline combinations. Based on this 2–19 division, binomial test revealed that the 95% and 99% C.I. for athletes whose choice for sub-technique was not affected by protocol but only by the speed-incline combination were 4–21% and 2–28%, respectively (the group applying different sub-techniques: 79–96% and 72–98%).

### Effect of protocol direction

The WR-c protocol did not show any hysteresis effect, whereas SPD-c showed a highly significant effect of around 1% incline (Table 1). There was no sex effect on change of direction.

**Table 1:** Incline (%) of sub-technique transition in two protocols for the initial (increasing incline) and reversed (decreasing incline) order.

Protocol	Direction	DP-DK	p	DS-DK	p
WC-r	Initial	5.44 ± 1.10	0.14	7.52 ± 1.50	0.72
	Reversed	5.13 ± 1.01		7.44 ± 1.09	
SPD-c	Initial	5.72 ± 1.28	<0.001	7.66 ± 1.04	<0.001
	Reversed	4.64 ± 1.14		6.82 ± 1.27	

The p-values are based on paired *t*-test. An extended table is available as supporting information file.

DP: double poling, DK: double poling with kick, DS: diagonal stride.

### Relationship with upper and lower body strength

**Fig 4:**
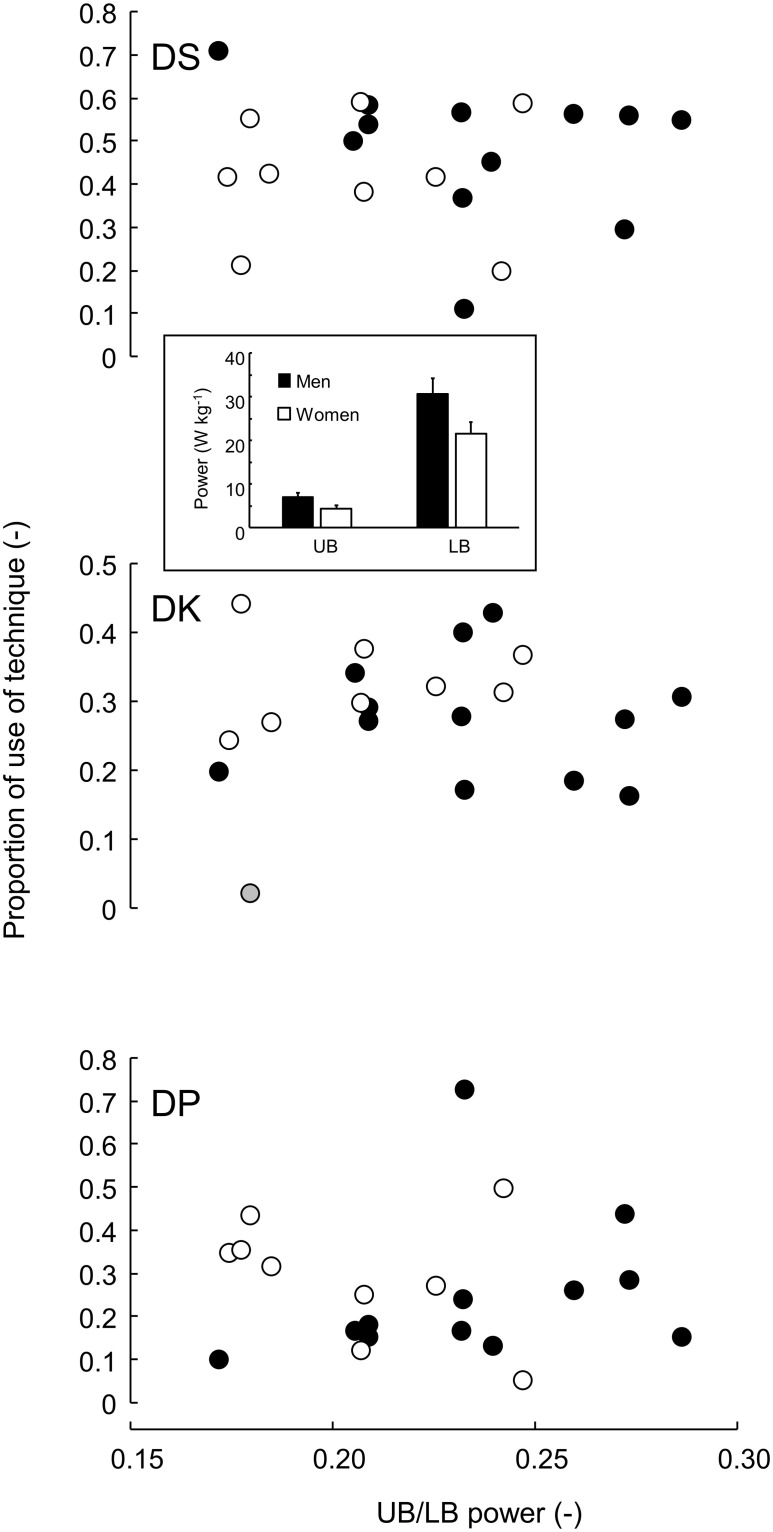
Proportional use of sub-technique against strength ratio. Proportional use as in Fig 3. Strength ratio is expressed as upper body (UB) / lower body (LB) Inset: Mean power in UB and LB for men and women. Bars indicate S.D. Black symbols: men; white symbols: women. Grey marker (for DK) is considered outlier (woman) (see text).

Both men and women had considerably higher LB than UB power (Fig 4 inset), leading to overlapping UB/LB power ratios for both sexes, but on average slightly lower in women (Fig 4 two-sample t-test: p = 0.042). This was not reflected in the use of sub-techniques, which did not show any sex effect (two-sample t-tests: p>0.36). Furthermore, except for one occasion, no significant relationships were found between upper—and lower body strength and their ratio on the one hand and use of any of the sub-techniques on the other (Fig 4). Only in women, a relationship between upper body strength and use of DK was significant (n = 9, r = 0.744, p = 0.021). Significance disappeared after removal of a potential outlier (for both strength and DK use).

## Discussion

The aim of this study was to investigate which external factors, speed, incline, or work rate, were the main determinant for sub-technique choice in classical roller skiing under moderate intensity conditions. In the constant work rate and constant speed condition, mostly incline rather than speed determined the choice of sub-technique, confirming earlier work [11]. However, in the constant incline condition, i.e., while skiing at one incline, different sub-techniques were applied. Under this condition, speed seems to play the major role for the choice of sub-technique, not work rate. While some differences were noted between men and women in this behavior, the general trends were the same for both sexes. Most athletes applied different sub-techniques at a particular speed-incline-work rate condition, depending on the test protocol, i.e., history of external condition. Hysteresis, i.e., order of change of condition, was identified for the constant speed protocol, but not for the constant work rate protocol. The proportional use of sub-technique was not associated in any manner with strength (power) of upper and lower extremities. It should be noted that our statistical analysis mostly identified the factors that did not function as a control variable for sub-technique shifts, because these variables at time of shift were clearly not the same for the three levels of the protocol (ANOVA: p-values = <0.001). The conclusion about if a variable is to be considered a control variable on the basis of non-significance is harder to verify by this statistical outcome only (type II error, power of the test—see ‘supporting material’). Our interpretation is therefore partly based on the process of elimination: if one factor clearly is not a control variable, the one that remains most likely is.

### Incline—Speed—Work rate

Overall, incline seems to control the sub-technique choice more than speed, while external work rate for the current conditions can almost be ruled out in this role. In this regard, it would be of interest to investigate the role of total work rate, or maybe even more so, total physiological load (metabolic rate) as a potential control variable. Our findings defy the notion that a single factor fully determines sub-technique choice. First, while incline appears to be a major control variable, during the constant incline conditions, most athletes used all sub-techniques. Furthermore, the vast majority of athletes chose a sub-technique for a particular incline-speed (and work rate) combination that depended on the protocol. Third, hysteresis effects were identified for the constant speed protocol. The overall image that is depicted from comparing the proportional use of sub-techniques depending on one condition parameter (Fig 3), indicates that besides incline, speed seems to be a control variable of importance. This applies especially to for the DS-DK shift in WC-r, for which both factors, in isolation, should be ruled out as a control variable (see Fig 2B). Recent data [23] revealed that during the Norwegian national championships all three sub-techniques were used at different inclines (~0% DP only; ~6% mixed and ~12% DS only), but at considerably higher speeds, and likely work rate, than in the current study. Clearly, not all combinations relevant for cross-country skiing were investigated in our study. Still, regardless the relatively low speeds (and work rates) applied here, the athletes were free to choose sub-technique at any time. Regarding incline, our findings (Fig 3A) agree very well with results from competition [23]. Thus also the comparison of the present study with [23] indicates that, in isolation, work rate does not seem to be a factor that has a strong impact on sub-technique choice. Although the current study provides the outcome of an extended ‘terrain’ of possible conditions compared to [11], the picture is still incomplete. Future studies in which these factors are made independent are warranted. In any way, the current results show that the rationale put forward by e.g., Pellegrini et al. [10] and Dahl et al. [13] regarding the minimal propulsion time that is required (i.e., speed as control variable), or by Ettema et al. [11] on the ability to use the body’s mechanical energy in DP by ‘falling’ on the poles (i.e., incline as control variable) cannot explain all preferences of sub-technique as a consequence of external condition.

In the current study, the period for one condition were much shorter (15 s) than in our previous study [11] (60 s). This was done to keep duration of the exercises short and may have given the changes of condition a more ‘continuous’ feel to the athletes. The comparable outcome of this study and [11] for the WR-c protocol might indicate that the duration of protocol and condition does not play a role of high importance.

### Hysteresis

Hysteresis was investigated only for incline, and thus the INC-c protocol was not included. As in our previous study [11], we did not detect hysteresis behavior (i.e., difference between initial vs reversed order) for sub-technique choice in the WR-c protocol. However, the present study revealed that hysteresis occurred for the SPD-c protocol. Earlier, we suggested that the incline sensitivity (1%) was not sufficient to detect hysteresis [11]. This is not confirmed in our current findings; the protocols had the same 1% incline sensitivity which apparently was sufficient to detect hysteresis in the SPD-c situation. The finding for SPD-c is in line with what is generally found in motor behavior and with theory of motor control [24]. Thus, the lack of hysteresis in the WR-c protocol most likely reflects a genuine characteristic that defies the general notion of hysteresis. Most studies that have investigated gait transitions, including hysteresis, used protocols where exercise intensity changes, e.g., gait on level ground at different speeds [25–27]. It is striking that when the intensity is kept constant (as in walking and running at changing speed and incline), hysteresis does not occur. This finding indicates that the perception of exertion plays a subtle role in the choice of sub-technique. It is possible that when this perception changes continuously, the effect by changing gait mode on the ‘cost’ of exercise is camouflaged to some degree. Thus, it will require a larger condition difference before the ‘benefit’ of changing gait mode is detected, i.e., showing more hysteresis. Obviously, the finding of the current study merely triggers this hypothesis but by no means verifies it.

### Performance capacity in upper and lower body

The current study did not find any relationship between preference of sub-technique and strength in upper and lower extremities. This was somewhat surprising and in disagreement with earlier findings [15] and the general notion that arm strength is required for successful DP. One can speculate about the reasons, but it is unlikely that the group was too homogeneous to detect any strength—sub-technique preference relationship (see Fig 4). The strength tests that were used do not mimic the ski movements perfectly but are believed to be more specific for cross-country skiing than standard strength tests [28, 29]. All skiing exercises were at moderate intensity and never challenging the athletes on performance. We suspect that this may be one of the reasons for lack of a relationship. In that respect, the two athletes that used one sub-technique in a given condition independent of history had close to mean values of upper-to-lower body strength ratio. In fact, this finding strengthens the outcome of the main part of the study is general, independent on an athlete’s strength profile, which may be related to sub-technique preference.

## Supporting information

S1 Data (XLSX)

S2 Data (XLSX)
